# Platelets link coagulation and complement in regulating murine placental vascular development

**DOI:** 10.1172/JCI200372

**Published:** 2026-07-23

**Authors:** Arno Smid, Lisa Schumann, Olga Oleshko, Ulrike Peters-Bernard, Kerstin Flächsig-Schulz, Melissa Whitehead, Emma Arndt, Korbinian Brand, Sonja Werwitzke, Andreas Klos, Bryan Paul Morgan, Wioleta M. Zelek, Andreas Tiede, Markus Abeln

**Affiliations:** 1 Institute of Clinical Biochemistry,; 2 Department of Haematology, Haemostasis, Oncology, and Stem Cell Transplantation,; 3 Institute of Clinical Chemistry and Central Laboratory, and; 4 Institute of Medical Microbiology and Hospital Epidemiology, Hannover Medical School, Hannover, Germany.; 5 Division of Infection and Immunity and UK Dementia Research Institute, School of Medicine, Cardiff University, Cardiff, United Kingdom.

**Keywords:** Immunology, Reproductive biology, Complement, Glycobiology, Platelets

## Abstract

During early pregnancy, maternal blood surrounds the embryo before the placenta is fully developed, requiring tight regulation of maternal blood flow into the placental vasculature. We identify placental microthrombi (PMTs) as essential structures guiding this process. PMTs contain platelets, coagulation factors, and complement proteins, and their formation depends on maternal platelet activation by thrombin through the protease-activated receptor 4 (PAR4). Deficiency of PAR4 abolished PMTs and caused excessive bleeding at the implantation site. C3 deficiency also led to increased bleeding events, indicating that complement activation contributes to thrombosis in the placental circulation. Conversely, dysregulated complement activation in CMP–sialic acid synthase–deficient (*Cmas^–/–^*) mice led to widespread thrombosis and failed placental development. Strikingly, platelet activation via PAR4 was necessary to localize complement activation to trophoblast surfaces, thereby coupling coagulation and complement in PMT formation. Depletion of maternal platelets mitigated complement-driven thromboinflammation in *Cmas^–/–^* pregnancies, restoring placental growth. These findings uncover a critical cooperation between platelets, coagulation, and complement in establishing maternal blood flow to the placenta. Successful pregnancy therefore requires not only activation but also tight regulation of these systems to balance necessary PMT formation with the prevention of pathological thrombosis.

## Abstract 2

Platelets, blood clotting, and immune proteins must balance perfectly to safely direct blood flow to the developing placenta during early pregnancy.

## Graphical abstract

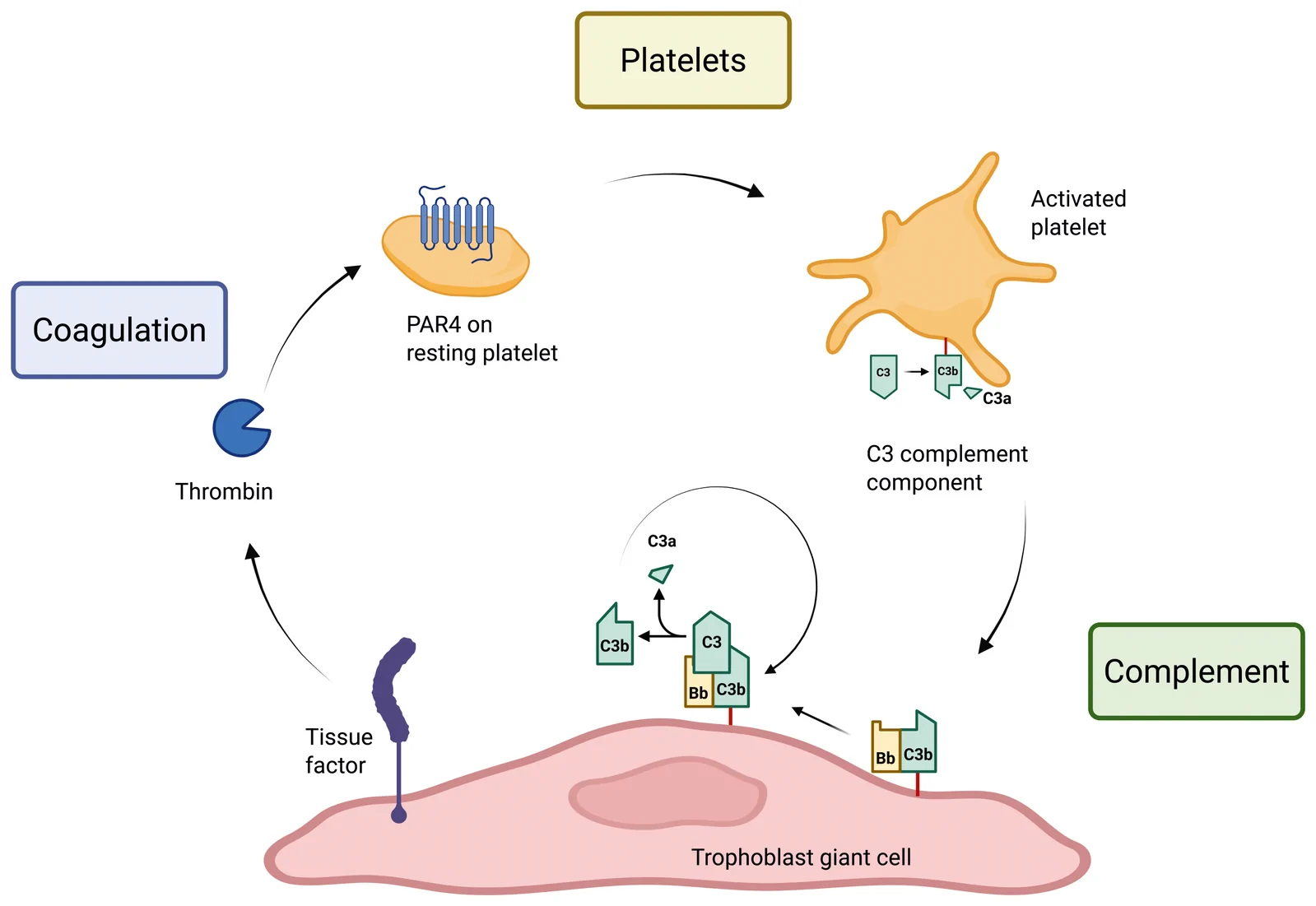

## Introduction

For a pregnancy to be successful, several challenges must be overcome, some of which appear to be mutually exclusive. On the one hand, the maternal and fetal blood must be in close proximity to ensure the fetus is adequately supplied with oxygen and nutrients; on the other hand, immunological shielding is essential to prevent an immune response against paternal antigens from the maternal immune system. Both mice and humans develop a hemochorial placenta, in which maternal blood is in direct contact with fetal trophoblasts, thus enabling an efficient supply of fetal tissues. This requires a variety of local and systemic immunological adaptations to allow close interaction of mother and embryo.

In the early stages of hemochorial pregnancy, before fetal blood circulation has developed, the embryo is surrounded by maternal blood. Even at this early stage, nutrients are actively transported toward the embryo by the extraembryonic tissue (1). However, rapid embryonic growth soon exceeds the capacity of this supply system. A recently published study showed that, by E9, the implantation site becomes severely hypoxic (2). During this period, the embryonic circulation develops, and from E10 onward, nutrient exchange between the fetal and maternal blood begins in the placenta (3). Maternal blood flow through the developing placenta must be tightly regulated and mechanisms of hemostasis and thrombosis appear to be involved in this regulation.

Trophoblast giant cells (TGCs), which form the outermost layer of the placenta at this stage, express potent procoagulant molecules like tissue factor (TF) (4), as well as anticoagulant proteins including endothelial protein C receptor (EPCR) and thrombomodulin (TM) (4). Loss of TF, EPCR, or TM is embryonically lethal around E9, highlighting the importance of a balanced pro- and anticoagulant system. Notably, embryonic lethality caused by loss of EPCR or TM can be partially rescued by rebalancing the mechanisms of thrombosis and hemostasis, for example by inhibiting other molecules involved in thrombin generation (e.g., factor VIII [FVIII]), or maternal platelet activation via deletion of the protease-activated receptor 4 (PAR4) (5, 6). Together, these findings demonstrate that, although TGCs have pronounced procoagulant properties of vital importance, excessive thrombosis must be prevented at the same time.

Interestingly, deficiencies in complement regulation produce phenotypes similar to those observed in TM- and EPCR-deficient animals. Loss of the complement receptor type 1–related gene Y protein (Crry) or of CMP–sialic acid synthase (CMAS) — the latter being essential for the biosynthesis of sialic acid–bearing glycans, which enables fully functional complement regulator factor H — leads to severe deficits in placental development and, ultimately, embryonic death (7–9). In both models, cellular damage is not driven by the formation of the membrane attack complex, and the precise cause of placental malformation remains unclear (7, 9). The temporal congruence of the phenotypes of mice with deficits in anticoagulation (TM and EPCR) and complement regulation (Crry and CMAS) suggests a close link between complement, coagulation, and the developing placenta.

In this study, we examined mouse models with defects in the coagulation or complement cascades, as well as platelet activation to explore mechanisms of thrombosis and hemostasis that are essential for successful placental development. Our data show that PAR4-mediated activation of platelets was essential for placental microthrombus (PMT) formation at the fetal-maternal interface. The absence of PMTs leads to severe bleeding events at mid-gestation. Proteins of the complement system are also present in these placental thrombi, and absence of C3 leads to increased placental bleeding. Using a complement-sensitive mouse model (CMAS^–^), we demonstrate that excessive complement activation resulted in increased thrombosis, a process very similar to the pathogenic process of microvascular thromboinflammation described in a diverse range of human diseases (10). Notably, loss of PAR4 diminished complement deposition on CMAS^–^ trophoblasts but did not rescue the development of the placenta. Only a severe reduction of the platelet count during pregnancy prevented complement-mediated thrombosis in CMAS-deficient pregnancies, suggesting that PAR4^–^ platelets were activated by other mechanisms at the fetal-maternal interface. Together our findings demonstrate that platelet and complement activation are crucial for preventing hemorrhage during pregnancy; however, platelet-mediated complement activation needs to be tightly controlled to prevent thrombosis and severe placental pathology.

## Results

### Microthrombosis and bleeding in the developing placenta.

**Figure 1:**
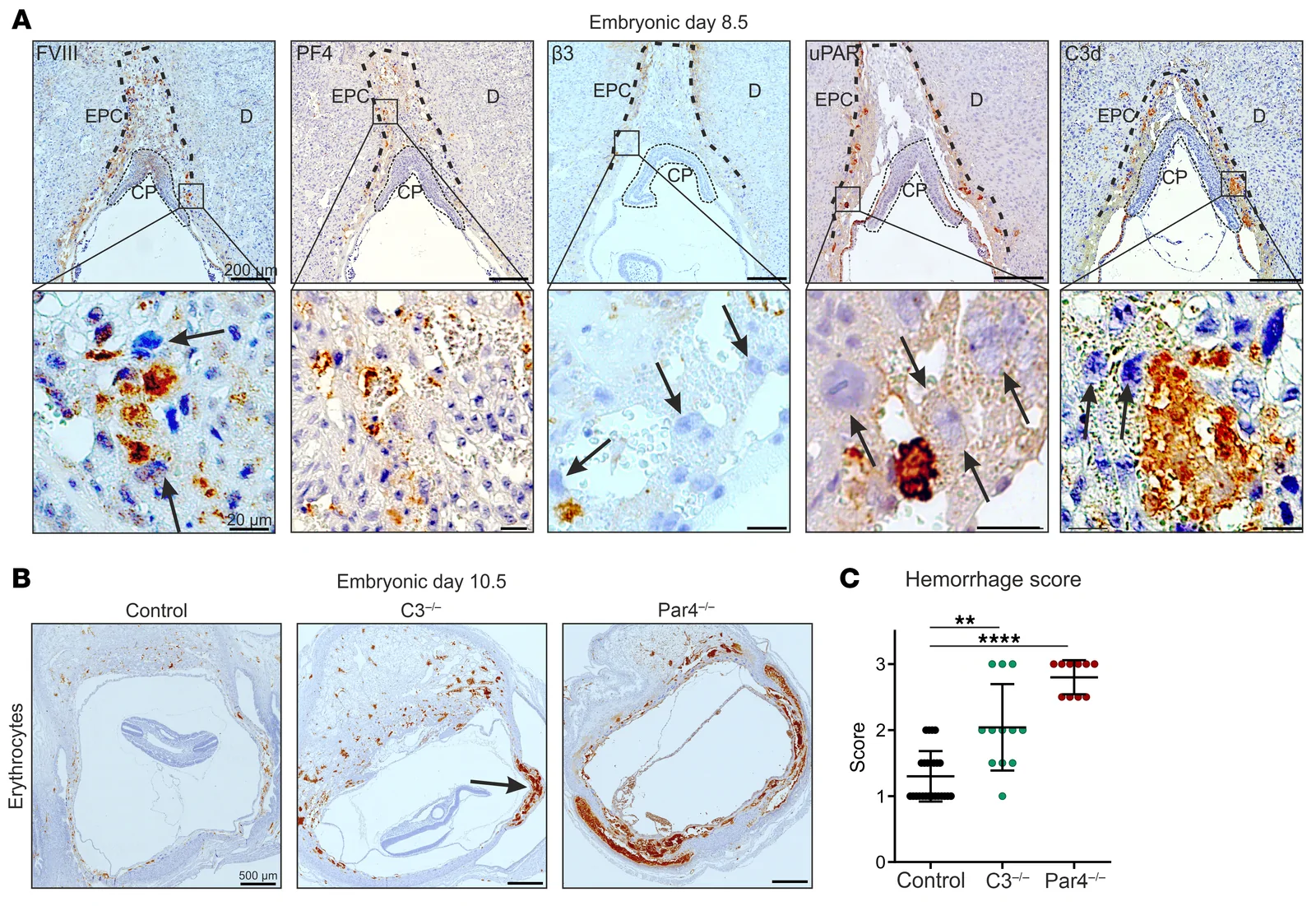
Coagulation and complement regulate maternal blood flow at the developing placenta. (**A**) Representative images of FVIII, PF4, β3, uPAR, and C3d immunostaining of placenta sections at E8.5. Arrows point to polyploid TGCs. For the number of individually analyzed implants, see Supplemental Table 1. EPC, ectoplacental cone; D, decidua. Scale bars: 200 μm (top panel) and 20 μm (enlarged insets in bottom panel). (**B**) Representative images of distribution of maternal erythrocytes (TER-119) at E10.5 in pregnancies of control, *C3^–/–^*, and *Par4^–/–^* mice. Arrow points to antimesometrial hemorrhage. Scale bars: 500 μm. (**C**) Hemorrhage score for the images in **B**, ranging from 1 (none to mild), 2 (intermediate), to 3 (severe). Data are presented as the mean ± SD. **P < 0.01 and ***P < 0.0001, by Kruskal-Wallis test. *n =* 25 controls from 3 pregnancies; *n =* 12 *C3^–/–^* mice from 2 pregnancies; *n =* 10 *Par4^–/–^* mice from 2 pregnancies.

In order for maternal blood to stop flowing around the implantation site and instead be directed through the blood vessels forming in the developing placenta, the blood flow must be blocked locally. Analysis of the boundary layer between trophoblasts and decidua on E8.5 revealed the presence of PMTs in the vicinity of TGCs on the mesometrial side of the placenta (Figure 1A). These PMTs contained coagulation proteins (e.g., FVIII), maternal platelets (detected through platelet factor PF4 and β3 integrin [β3]), and urokinase-type plasminogen activator receptor (uPAR), which is part of the fibrinolytic system. Interestingly, PMTs contained not only platelets and coagulation proteins, but also the complement protein C3. This is shown by reactivity for the C3 activation product C3d (Figure 1A).

We then investigated the influence of platelets and the complement system on the direction of blood flow at the developing placenta. At E10.5, when maternal blood flow through the placenta is generally established, the placenta of control animals showed few maternal erythrocytes at the implantation site near the antimesometrial area, indicating a functional barrier and established blood flow through the placenta (Figure 1B). In contrast, C3^–^ or PAR4^–^ pregnancies showed increased deposition of erythrocytes, indicating failed guidance of the blood flow. Using a score ranging from 1 (mild) to 3 (severe), we found significantly more hemorrhaging in C3^–^ and PAR4^–^ animals than in the control animals (Figure 1C).

### PAR4-mediated platelet activation is crucial for PMT formation.

**Figure 2:**
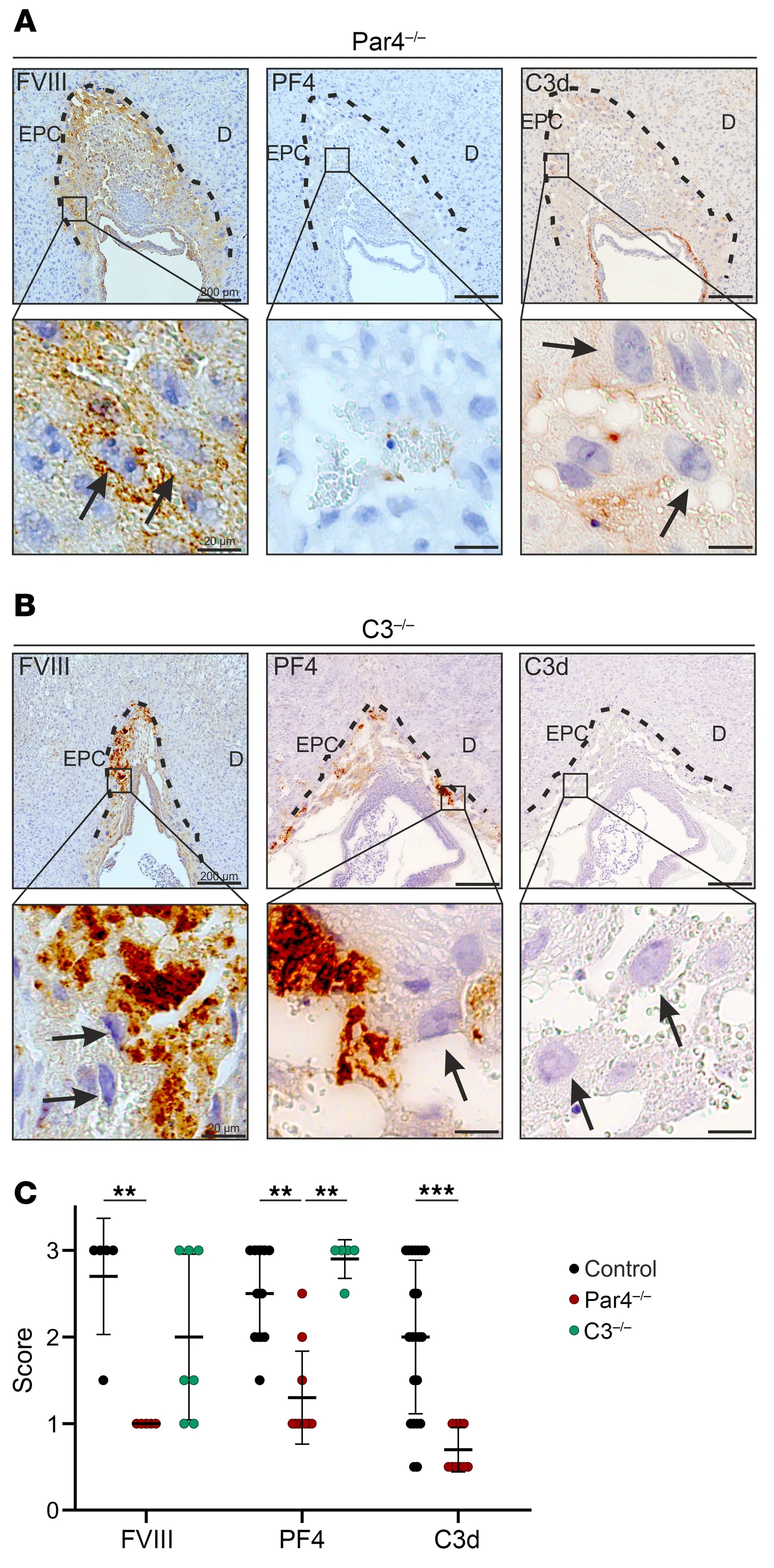
PAR4-mediated platelet activation is crucial for thrombus formation at the placenta. (**A**) Representative images of FVIII, PF4 and C3d immunostaining of *Par4^–/–^* placenta sections at E8.5. Arrows point to polyploid TGCs. Scale bars: 200 μm (top panel) and 20 μm (enlarged insets in bottom panel). (**B**) Representative images of FVIII, PF4, and C3d immunostaining of C3*^–/–^* placenta sections at E8.5. Arrows point to polyploid TGCs. Scale bars: 200 μm (top panel) and 20 μm (enlarged insets in bottom panel). For the number of individually analyzed implants, see Supplemental Table 1. (**C**) IHC scoring of C3d, FVIII, and PF4 in PMTs from control, *Par4^–/–^*, and *C3^–/–^* placenta sections at E8.5. The score ranges from 1 to 3 for FVIII and PF4 and from 0 to 3 for C3d. Data are presented as the mean ± SD. **P < 0.01 and ***P < 0.0001, by Kruskal-Wallis test (FVIII and PF4) and Mann-Whitney *U* test (C3d).

As the absence of both C3 and PAR4 leads to deficits in guidance of maternal blood, we examined the composition of PMTs at the fetal-maternal interface in C3^–^ and PAR4^–^ animals at E8.5. In placentas from PAR4^–^ mothers, FVIII was no longer enriched in clots, but diffusely associated with TGCs (Figure 2, A and C). Moreover, only very few platelets (PF4) and hardly any reactivity for C3d were found (Figure 2, A and C), indicating a complete absence of PMTs. In C3^–^ pregnancies, all the aforementioned components were present except for C3 (Figure 2, B and C). These data imply that PAR4-mediated platelet activation at the fetal-maternal interface is essential for the formation of PMTs, which substantially contribute to regulation of maternal blood flow at the developing placenta. In the absence of C3, PMT formation was observed, but it was unable to sufficiently guide blood flow to the placenta and prevent antimesometrial hemorrhage.

### Loss of placental complement regulation results in excessive thrombosis.

**Figure 3:**
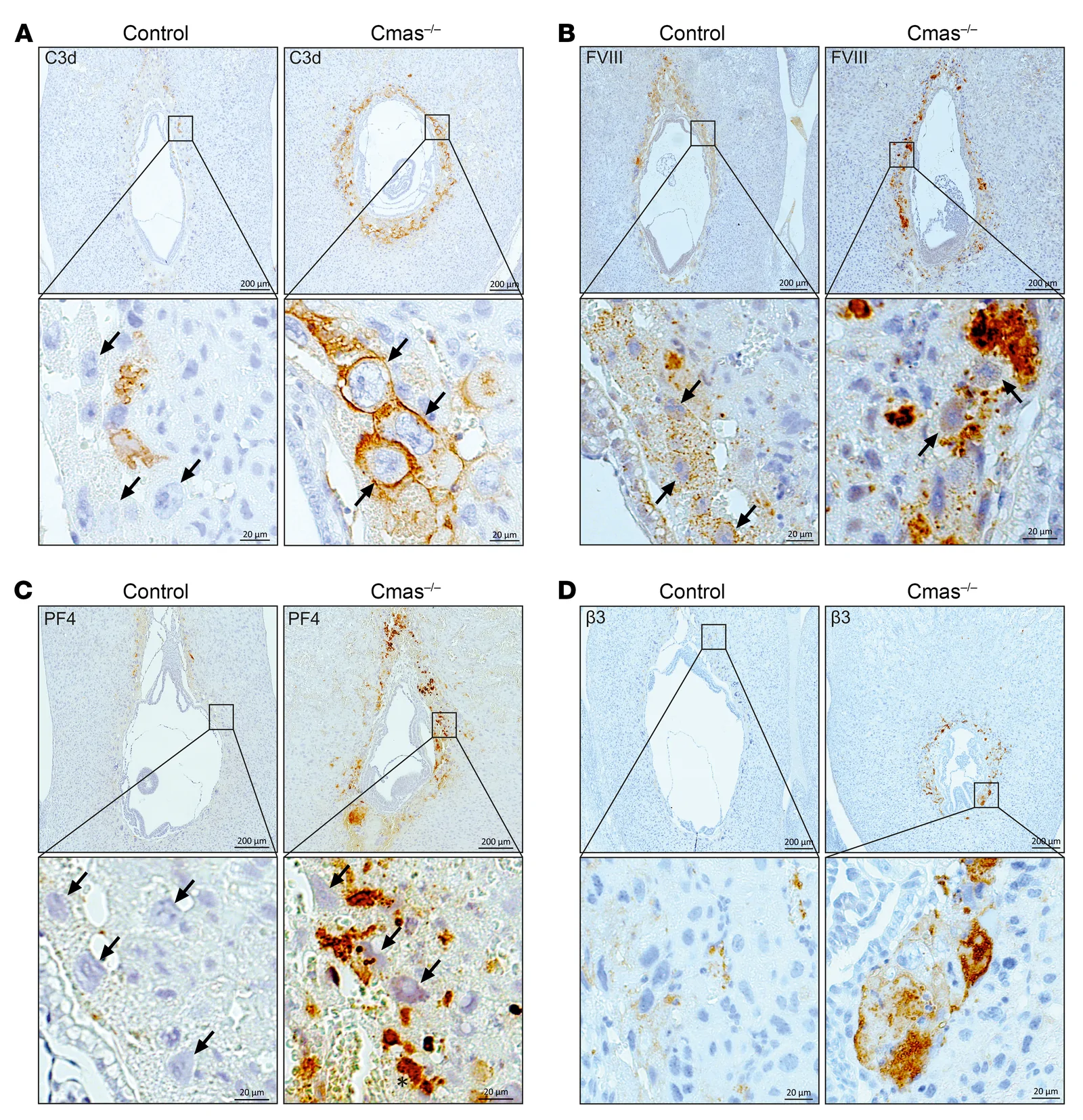
Loss of placental complement regulation results in excessive thrombosis. (**A**–**D**) Representative images of C3d (**A**), FVIII (**B**), PF4 (**C**), and β3 (**D**) immunostaining of control and Cmas^–/–^ placenta sections at E8.5. Arrows point to polyploid TGCs. Scale bars: 200 μm (top panel) and 20 μm (enlarged insets in bottom panel). For the number of individually analyzed implants, see Supplemental Table 1.

Since excessive complement activation is associated with human pregnancy complications and mouse models with impaired complement regulation show severe placental developmental deficits, we asked if platelet activation contributes to complement-mediated placental malformations. As we have previously demonstrated, loss of sialoglycans, due to impaired activation of sialic acid (*Cmas^–/–^*), results in excessive activation of the maternal complement system and pregnancy loss (7) (Figure 3A). Investigation of coagulation proteins at the fetal-maternal interface of *Cmas^–/–^* mice revealed that FVIII^+^ thrombi form around the implantation site with increased numbers and size (Figure 3B). Likewise, the implantation site of *Cmas^–/–^* animals revealed a pronounced infiltration with maternal platelets (Figure 3, C and D). To determine whether this pathological thrombosis depends on activation of the complement system, we analyzed *Cmas^–/–^* embryos on a C3^–^ background. C3 deficiency rescued the placental developmental deficit (Supplemental Figure 1; supplemental material available online with this article; https://doi.org/10.1172/JCI200372DS11). The formation of PMTs, the deposition of FVIII, and maternal platelets were similar to what we observed in normal controls again (Supplemental Figure 1). These data demonstrate that loss of complement regulation can cause excessive thrombosis, which can be corrected by C3 deletion.

### C5b-9 and anaphylatoxin signaling do not contribute to thrombosis in Cmas^–/–^ pregnancies.

Formation of the membrane attack complex (MAC or C5b-9) is the terminal step in the complement cascade, and several studies have demonstrated procoagulant properties of C5b-9 (11, 12). However, interruption of the complement cascade already at the step of C5 activation had no effect on excessive thrombosis and pregnancy loss in *Cmas^–/–^* pregnancies (Supplemental Figure 2, A–D). Of note, the pronounced mesometrial C3d and PF4 reactivity in controls under C5 blockade indicate that C5b-9 was not involved in PMT formation, further strengthening the point that platelet activation and C3 are the main drivers of clot formation at the developing placenta.

We also addressed the potential role of the anaphylatoxins C3a and C5a, as these inflammatory mediators can contribute to a procoagulant environment (13). However, *Cmas^–/–^* mice on a C3aR/C5aR1^–^ background showed no improvement in complement activation, thrombosis, or placental development (Supplemental Figure 3, A–C).

Together, neither the terminal complement cascade nor anaphylatoxin signaling was involved in the excessive thrombosis observed in CMAS-deficient pregnancies. The activation of platelets and C3 was sufficient to cause thrombosis.

### Complement activation decreases trophoblastic TM.

**Figure 4:**
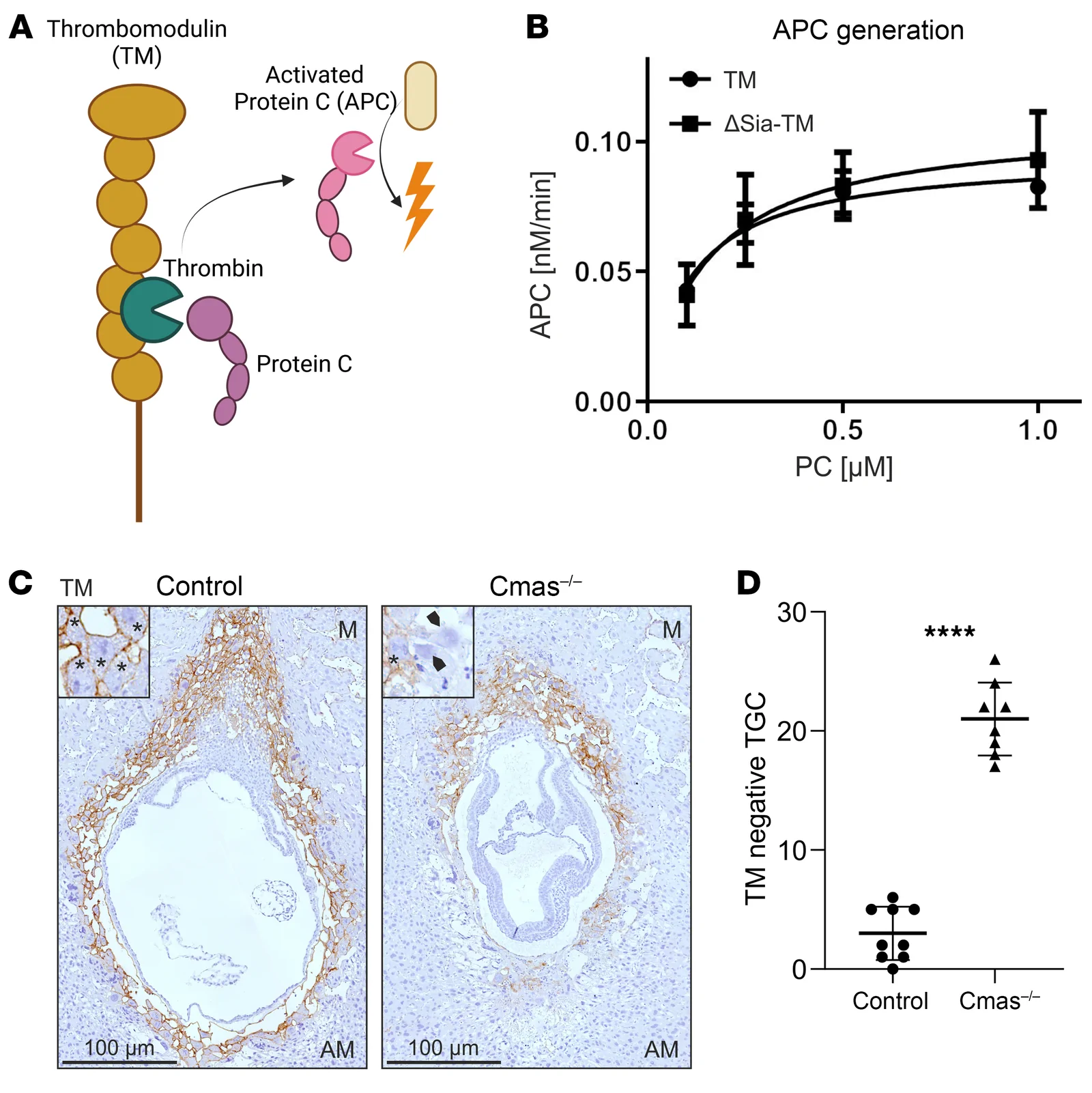
Complement activation results in decreased TM levels. (**A**) Schematic representation of the APC generation assay. The TM-thrombin complex proteolytically activates protein C to APC. APC then proteolytically cleaves a chromogenic reporter molecule. Created with BioRender. (**B**) APC generation kinetics of thrombin in a 1:1 ratio with TM and ΔSia-TM. Data are presented as the mean ± SD of duplicates. (**C**) Representative images of TM immunostaining of control and *Cmas^–/–^* placenta sections at E8.5. Inset: asterisks mark TM^+^ and arrowheads point to TM^–^ TGCs. M, mesometrial region of the decidua; AM, antimesometrial region of the decidua. Scale bars: 100 μm. Insets: original magnification ×3.66. For the number of individually analyzed implants, see Supplemental Table 1. (**D**) Quantification of TM^–^ TGCs in control and *Cmas^–/–^* pregnancies at E8.5. Data are presented as the mean ± SD. ****P < 0.0001, by unpaired 2-tailed *t* test.

Another potential link between dysregulated complement activation and thrombosis might be the endothelial surface protein TM, which activates protein C, which then inactivates the coagulation factors Va and VIIIa. TM is expressed on TGCs and — similar to *Cmas^–/–^* embryos — TM-deficient embryos die at E8.5 (14). TM forms a complex with thrombin on the cell surface. At first, we aimed to determine whether desialylated TM (ΔSia-TM), as it occurs on the cell surface of CMAS^–^ TGCs, has impaired capacities in activated protein C (APC) generation. To generate ΔSia-TM, recombinant TM was incubated with neuraminidase-coupled beads (Supplemental Figure 4A), and we confirmed its enzymatic activity in a colorimetric assay (Supplemental Figure 4B). TM and ΔSia-TM were then used together with thrombin in an APC generation kinetic assay that measures the proteolytic activity of APC according to published methods (Figure 4A) (15). Both TM glyco-variants yielded very similar amounts of APC, indicating that the sialylation status of TM did not affect its activity in the generation of APC (Figure 4B and Supplemental Figure 4, C and E). Since TM shedding is known to occur in the context of inflammatory processes (16), we next performed IHC to analyze the presence of TM in *Cmas^–/–^* trophoblasts. While the implants in control mice showed pronounced levels of TM in TGCs along the entire fetal-maternal interface, CMAS^–^ mice had decreased reactivity for TM, especially on the antimesometrial site (Figure 4C). Also, the presence of TM^–^ TGCs was significantly higher in CMAS^–^ implants compared with controls (Figure 4D). This suggests that complement-related inflammatory processes decreased the levels of TM on the surface of trophoblastic cells, which in turn promoted thrombosis at the fetal-maternal interface.

### Platelet activation stimulates C3 deposition of Cmas-deficient trophoblasts.

**Figure 5:**
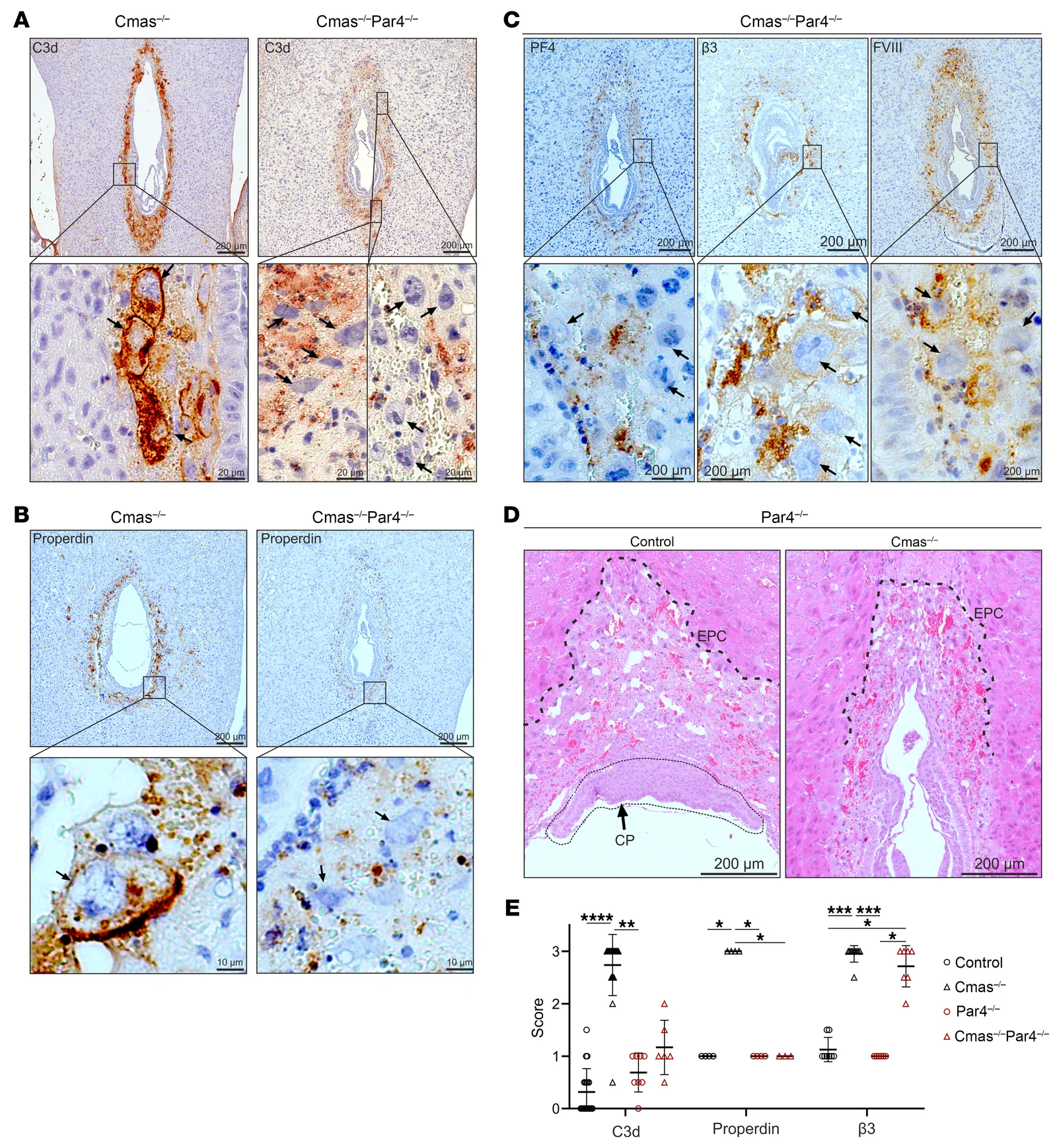
PAR4-mediated platelet activation drives C3 deposition on trophoblasts, but is not solely responsible for platelet recruitment to the fetal-maternal interface. (**A** and **B**) Representative images of C3d (**A**) and Properdin (**B**) immunostaining of *Cmas^–/–^* and *Cmas^–/–^* *Par4^–/–^* placenta sections at E8.5. Arrows point to polyploid TGCs. Scale bars: 200 μm (top panels in **A** and **B**) and 20 μm (enlarged insets in bottom panel in **A**); 10 μm (enlarged insets in bottom panel in **B**). (**C**) Representative images of PF4, β3, and FVIII immunostaining of *Cmas^–/–^* *Par4^–/–^* placenta sections at E8.5. Arrows point to polyploid TGCs. Scale bars: 200 μm. (**D**) H&E staining of *Cmas^–/–^* implants at E8.5 in *Par4^–/–^* mothers. Scale bars: 200 μm. For the number of individually analyzed implants, see Supplemental Table 1. (**E**) IHC scoring of C3d, Properdin and β3 of control, *Cmas^–/–^*, *Par4^–/–^*, and *Cmas^–/–^ Par4^–/–^* placenta sections at E8.5. The score ranges from 0 to 3 for C3d and from 1 to 3 for properdin and β3. Data are presented as the mean ± SD. *P < 0.05, **P < 0.01, ***P < 0.001, and ***P < 0.0001, by Kruskal-Wallis test.

As PAR4-mediated platelet activation was required for PMTs and complement activation, we also investigated the role of PAR4 in CMAS^–^ pregnancies. Indeed, we found that C3d deposition on the cell surface of CMAS^–^ TGCs was absent in PAR4^–^ pregnancies (Figure 5, A and E). However, a diffuse reactivity of C3d could still be observed in the vicinity of these cells. Analysis of the alternative complement pathway stabilizer properdin revealed a very similar picture. Properdin was localized to the cell surface of CMAS^–^ TGCs in *Par4^+/+^*, but not in *Par4^–/–^*, pregnancies (Figure 5B). Instead, we observed diffuse deposition of properdin, similar to C3d, in *Cmas^–/–^ Par4^–/–^* animals. IHC analysis for PF4 and β3 showed that *Par4^–/–^* maternal thrombocytes were still recruited to the fetal-maternal boundary layer in *Cmas^–/–^ Par4^–/–^* pregnancies (Figure 5C). Although we found no FVIII^+^ thrombi, we observed generally increased reactivity to FVIII in *Cmas^–/–^ Par4^–/–^* pregnancies (Figure 5C). Notably, despite the reduced complement activation on the cell surface, there was no improvement in placental development in *Cmas^–/–^ Par4^–/–^* pregnancies, as evidenced by the absence of the chorionic plate (CP) (Figure 5D). It can be concluded that the absence of PAR4-mediated platelet activation prevented the deposition of C3 on the surface of Sia^–^ cells; however, *Par4^–/–^* platelets and coagulation factors were still abundant in the *Cmas^–/–^* pregnancies/implants, and placental development remained severely disturbed.

### Depletion of maternal platelets mitigates excessive thrombosis and improves placental development in Cmas^–/–^ pregnancies.

**Figure 6:**
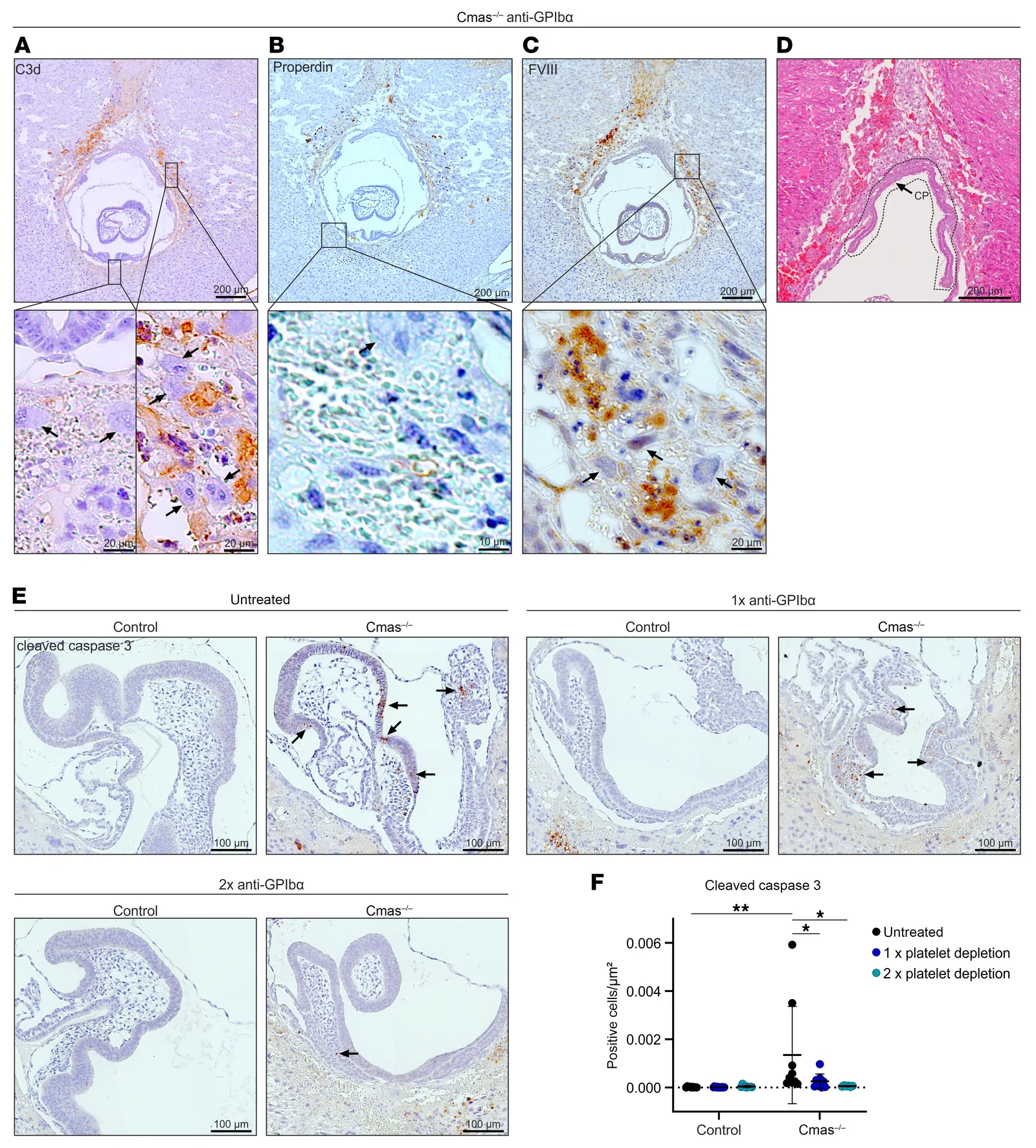
Platelet depletion mitigates excessive thrombosis and developmental deficits of the placenta. (**A**–**C**) Representative images of C3d (**A**), properdin (**B**), and FVIII (**C**) immunostaining of Cmas*^–/–^* implant sections at E8.5 in anti-GPIbα–treated mothers. Platelets were depleted by i.p. injection of 200 μg anti-GPIbα (R300 Emfret Analytics) at E2.5 and E5.5 in 3 individual experiments. Arrows point to polyploid TGCs. Scale bars: 200 μm (top panels in **A**–**C**), 20 μm (enlarged insets in bottom panels in **A** and **C**), and 10 μm (bottom panel in **B**). (**D**) H&E staining of *Cmas^–/–^* implants at E8.5 in anti-GPIbα–treated mothers. Scale bar: 200 μm. For the number of individually analyzed implants, see Supplemental Table 1. (**E**) Representative images of cleaved caspase 3 immunostaining of control and *Cmas^–/–^* implant sections at E8.5 in anti-GPIbα–treated mothers. Platelets were depleted by i.p. injection of 200 μg anti-GPIbα (R300, Emfret Analytics) at E4.5 or E2.5 and E5.5 in 3 individual experiments. Arrows point to representative cleaved caspase 3^+^ cells. Scale bars: 100 μm. (**F**) Quantification of cleaved caspase 3^+^ cells/μm^2^ of the embryo proper. Data are presented as the mean ± SD. *P < 0.05 and **P < 0.01, by 2-way ANOVA with multiple comparisons.

To investigate this hypothesis further, maternal platelets were depleted in the maternal circulation by anti-GPIbα injections during pregnancy. Platelet depletion abolished the deposition of C3d on TGCs and reactivity with properdin (Figure 6, A and B). The diffuse C3d and properdin reactivity still observed in PAR4^–^ pregnancies was markedly reduced upon platelet depletion (Figure 6, A and B). Similar to controls (Figure 1A and Figure 3B), FVIII reactivity in *Cmas^–/–^* mice was only found in PMTs at the mesometrial side of the fetal-maternal interface (Figure 6C). Moreover, the development of the placenta was also markedly improved in CMAS^–^ implants in platelet-depleted mothers (Figure 6D). Improvements in extraembryonic tissues were also accompanied by improved development of the embryo proper. The insufficient supply caused by extraembryonic defects in CMAS^–^ pregnancies resulted in increased apoptosis (cleaved caspase 3^+^ staining) in the fetus (Figure 6, E and F). Even a single depletion of maternal platelets during pregnancy resulted in fewer apoptotic cells, a reduction that was further improved by a second depletion.

These data strongly suggest that — despite the importance of PAR4 activation of platelets in the formation of PTMs — platelets provided a surface for ongoing activation of complement and coagulation on the *Cmas^–/–^* background that was only rescued with platelet depletion.

### PAR4-deficient platelets can still be activated and contribute to thrombosis in Cmas^–/–^ pregnancies.

**Figure 7:**
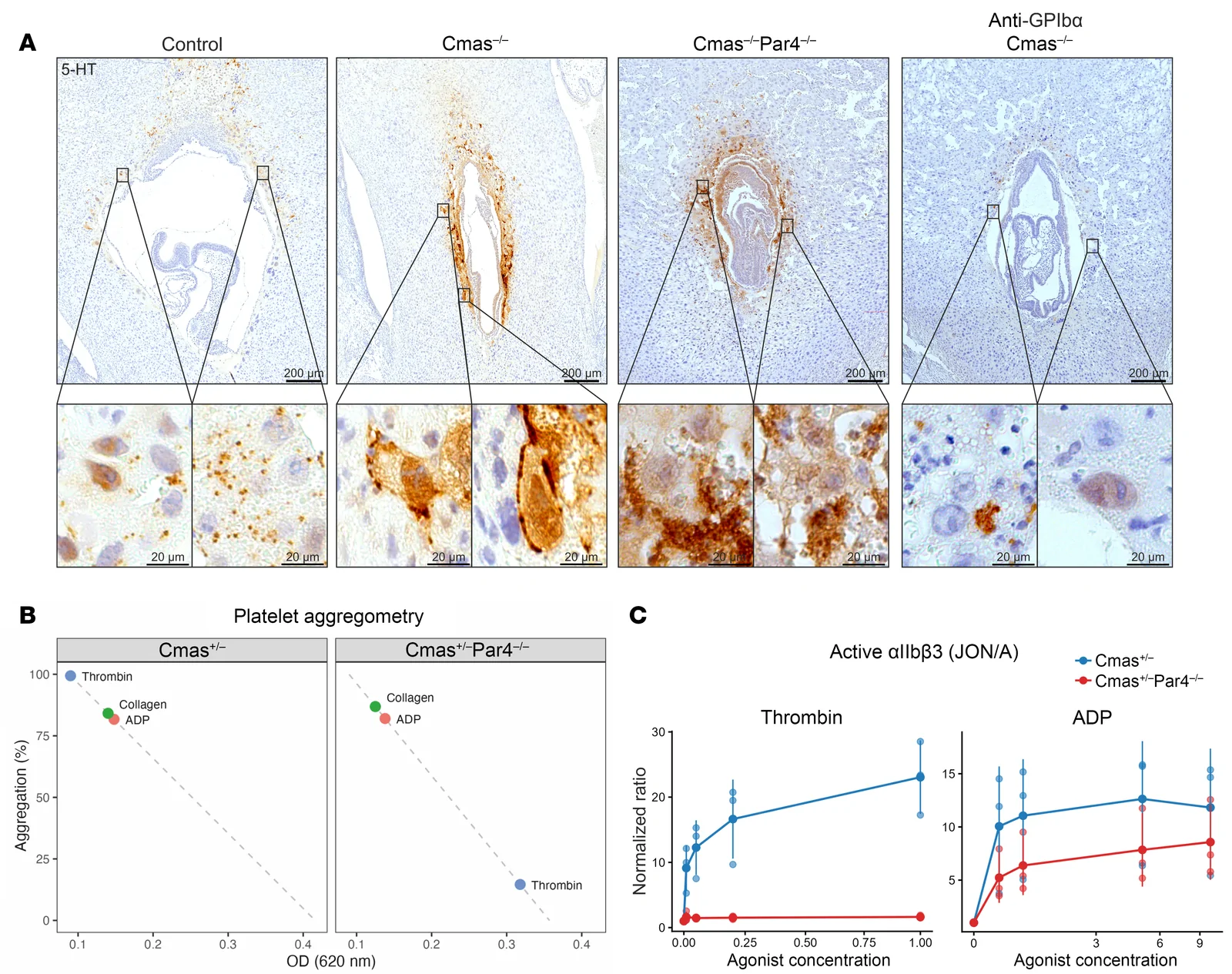
*Par4^–/–^* platelets at the fetal-maternal interface can be activated via thrombin-independent pathways. (**A**) Representative images of 5-HT immunostaining of *Cmas^–/–^* implant sections at E8.5 in control, *Par4^–/–^* mothers, and anti-GPIbα–treated mothers. Scale bars: 200 μm and 20 μm (enlarged insets in bottom panel). For the number of individually analyzed implants, see Supplemental Table 1. (**B**) Platelet aggregometry of PRP from *Cmas^+/–^* and *Cmas^+/–^ Par4^–/–^* mice subjected to thrombin, ADP, or collagen stimulus. (**C**) Flow cytometric analysis of αIIbβ3 integrin activation by thrombin or ADP stimulus in platelets from *Cmas^+/–^* and *Cmas^+/–^ Par4^–/–^* mice. Data are presented as the mean ± SD.

The substantial improvement in phenotype observed in pregnancies involving platelet depletion, compared with PAR4^–^ animals, suggests that platelet activation may still be occurring in the latter. To investigate this, we analyzed the presence of serotonin (5-HT), which is stored in platelet-dense granules and released upon activation. In control animals, we found small 5-HT^+^ particles that were approximately the size of platelets (Figure 7). These were most likely nonactivated platelets in which 5-HT was still stored in dense granules. Interestingly, a few TGC nuclei were also positive for 5-HT. We observed a very high reactivity for 5-HT in *Cmas^–/–^* embryos along the entire implantation site, suggesting release from activated platelets. A strong presence of 5-HT was also still evident at the fetal-maternal interface in *Par4^–/–^* animals. A completely different picture emerged in platelet-depleted pregnancies. Apart from the 5-HT^+^ TGC cell nuclei, which were also observed in the control animals, 5-HT was virtually absent. These data indicate that Par4^–^ platelets were still being activated at the fetal-maternal interface of CMAS^–^ pregnancies. To analyze which routes of activation are functional in *Cmas^+/–^* *Par4^–/–^* platelets, we conducted platelet aggregation and activation studies. As expected, thrombin induced aggregation of *Cmas^+/–^*, but not *Cmas^+/–^* *Par4^–/–^*, platelets. However, collagen and adenosine diphosphate induced the aggregation of both *Cmas^+/–^* and *Cmas^+/–^* *Par4^–/–^* platelets. Activation of integrin αIIbβ3, a key step in platelet activation, was induced by thrombin only in *Cmas^+/–^* platelets, while its induction by ADP was preserved in *Cmas^+/–^* *Par4^–/–^* platelets (Figure 2C, Supplemental Figure 5). These data reflect the well-known functional redundancy in platelet activation pathways and explain why PAR4 deficiency was less effective than platelet depletion in preventing complement activation and thrombosis during placental development in the CMAS-KO.

## Discussion

In this study, we investigated the regulated formation of PMTs, which are essential for directing maternal blood flow into the developing placenta. Our findings demonstrate that deficiencies in complement (*C3^–/–^*) or platelet activation (*Par4^–/–^*) resulted in excessive placental bleeding. This underscores the necessity of both systems in early pregnancy. Additionally, our findings reveal that PAR4-mediated platelet activation was necessary to localize C3 activation to PMTs, thereby linking coagulation and complement in placental development. Conversely, excessive complement activation in sialoglycan-deficient animals resulted in pathological thrombosis. This phenotype could be reversed by modulating platelet activity.

The role of PAR4 in linking coagulation and complement activation on TGCs is particularly intriguing. Platelet activation through PAR4 occurs upon cleavage of the receptor by thrombin, the central enzyme of the coagulation cascade. Thrombin generation in early pregnancy appears to rely primarily on the extrinsic pathway, since deficiency of TF, factor X, factor V, or prothrombin results in embryonic lethality around E9-E10 (17–20). In contrast, loss of intrinsic pathway components (including factors VIII, IX, XI) — or von Willebrand factor — does not impair embryonic survival, consistent with their dispensability for placental development (21–24). TGCs strongly express TF, and local activation of the TF/FVIIa/FX pathway provides a source of thrombin that activates platelets via PAR4 (4). Once activated, platelets contribute to thrombosis by exposing a procoagulant phospholipid surface, supplying coagulation factors, and releasing granule contents that increase the local calcium concentration. Our findings further demonstrate that PAR4-mediated platelet activation was required for complement C3 activation and that deficiency of either PAR4 or C3 resulted in excessive bleeding. So far, it has only been demonstrated that C3-deficient mice have an increased tail-bleeding time (25). Together, these data suggest that TF-driven coagulation on TGCs must be amplified by platelet and complement activation to enable proper PMT formation.

On the other hand, the interplay between coagulation, platelets, and complement required tight regulation to prevent pathological outcomes. In CMAS deficiency, factor H–mediated inactivation of C3 was impaired, leading to excessive complement activation and thrombosis — a process that can be called thromboinflammation — in the developing placenta. This pathological phenotype was partially rescued by either PAR4 deficiency and, even more completely, by maternal platelet depletion, demonstrating that platelets were necessary for C3 deposition and complement-driven thrombosis at the maternal-fetal interface. These findings highlight that, while platelet-driven complement activation is critical for proper PMT formation and placental hemostasis, dysregulation of this crosstalk can rapidly shift the balance toward excessive thromboinflammation. The procoagulant environment of the placenta is also sensitive to excessive coagulation reactions, which is demonstrated in mice with deficiency in TM or the EPCR; these mice die in utero around the same time as *Cmas^–^* and *Crry^–^* mice (7, 8).

The mechanisms described in this study are likely relevant to human disease. Platelet-derived C3 has been shown to contribute to inflammation in human influenza virus infection (26), and activated platelets can serve as a surface for complement activation on other cells (27, 28). In systemic lupus erythematosus, platelet activation collaborates with antiphospholipid antibodies to drive complement activation (29). Conversely, complement inhibition rescued a patient with severe coagulation and platelet activation in the context of vaccine-induced thrombotic thrombocytopenia (30). Excessive complement activation has also been linked to pregnancy complications, including preeclampsia (31), and has been observed in patients with systemic lupus erythematosus (SLE) who experience adverse pregnancy outcomes (32). Genetic polymorphisms in complement regulatory proteins, including factor H, CD46, and C4b-binding protein, which reduce expression or function, are associated with increased risk of severe preeclampsia and recurrent pregnancy loss (33, 34). Proteomics analyses further show that complement and coagulation proteins are highly dysregulated in early-onset, severe preeclampsia compared with controls, underscoring the close interplay of these pathways in adverse pregnancy outcomes (35).

Our data suggest that excessive complement activation contributes to pathological thrombosis in the placenta and can be mitigated by C3 deficiency or platelet depletion. Notably, deficiency in PAR4, unlike anti-GPIbα platelet depletion, did not completely prevent C3 activation in CMAS-deficient pregnancies, indicating that platelets activated through other pathways — such as ADP or collagen pathways — can also support complement activation. In the clinical setting, combination therapy with aspirin and low-molecular-weight heparin is the standard of care to prevent pregnancy loss in patients with antiphospholipid antibodies, but it is not always effective (36). Our findings suggest that targeting additional platelet activation pathways, together with inhibition of the alternative complement pathway, may represent a promising strategy for future clinical studies aimed at preventing placental thrombosis and pregnancy loss (37).

While our study provides new insight into the intricate balance of coagulation, platelet, and complement activation during placental development, several limitations should be acknowledged. First, although we demonstrate that platelet activation via PAR4 was required for C3 activation and PMT formation, the precise molecular interactions between TGCs and platelets remain unclear, and the mechanism by which platelets promote complement activation is not fully resolved. Second, we did not directly address the molecular mechanisms by which C3 activation or deposition contributes to placental thromboinflammation. Our data suggest that inhibition of C3a and C5a receptors or downstream complement/MAC activation does not prevent excessive thrombosis. While C3b-dependent amplification of complement and coagulation has been described in other contexts (38), the exact molecular mechanism in the placental environment remains to be elucidated. Third, our findings are based on mouse models, and species-specific differences in platelet and complement biology may limit direct extrapolation to humans. Finally, a limitation of this study is the relatively small sample size, which is common in exploratory basic research but may limit statistical power and generalizability, and although we used genetic and depletion approaches to manipulate platelets and complement, these interventions do not fully capture the subtleties of temporal and spatial regulation in normal pregnancy. Future studies using advanced imaging, cell-specific reporters, and human placental models will be required to dissect these interactions in greater detail.

In summary, our study reveals that the tightly coordinated activation of platelets, coagulation, and complement is essential for proper PMT formation and placental development. Disruption of this balance, as seen with PAR4 or CMAS deficiency, leads to pathological bleeding or thrombosis, highlighting the interdependence of these pathways. Our findings provide mechanistic insight into how dysregulated thromboinflammation may contribute to pregnancy complications and suggest that targeted modulation of platelet and complement activity could represent a promising avenue for therapeutic intervention. By illuminating these fundamental processes, our work lays the groundwork for future studies to better understand conditions of adverse pregnancy outcomes and their prevention.

## Methods

### Sex as a biological variable.

Only female animals were included in this study because the investigated phenotype is pregnancy related and therefore biologically restricted to females.

### Mice.

*Cmas^–/–^* mice (7, 39) were backcrossed for 6 generations on a NMRI background. *Cmas^–/–^* embryos were compared with *Cmas^+/+^* or *Cmas^+/–^* littermates controls.

*Cmas^+/–^ C3^–/–^* mice were generated by crossing *Cmas^+/–^* animals with the C3-depleted strain *B6* 129S4-C3^tm1Crr^/J. Upon 4 backcrosses with NMRI mice, heterozygous *Cmas*-KO mice on a homozygous *C3*-KO background (*Cmas^+/–^ C3^–/–^*) were obtained (stock *C3^tm1Crr^-Cmas^tm3^*). The following primers were used: ms C3geno forward, 5′-ATCTTGAGTGCACCAAGCC-3′; ms C3wt reverse, 5′-GGTTGCAGCAGTCTATGAAGG-3′; and ms C3mt reverse, 5′-GCCAGAGGCCACTTGTGTAG -3′. The PCR program included 3 steps (98°C for 15 seconds, 64.7°C). *B6*-*C3ar1^tm1Raw^-C5ar1^tm1Cge^* mice were provided by the Klos Laboratory (Hannover, Germany). Upon 4 backcrosses with NMRI mice, heterozygous *Cmas*-KO mice on a homozygous *C3aR C5aR1*-KO background (*Cmas^+/–^ C3aR^–/–^ C5aR1^–/–^*) were obtained (stock *C3ar^tm1Raw^-C5ar^tm1Cge^Cmas^tm3mhhTg15^*). The following primers were used: EW33 (5′-TACAATATAGTCAGTTGGAAGTCAGCC-3′), EW34 (5′-TGGGCTCTATGGCTTCTGAGGCGGAAAG-3′), and EW35 (5′-GAGAATCAGGTGAGCCAAGGAGAA-3′). The PCR program included 3 steps (98°C for 15 seconds, 64.3°C for 30 seconds, 72°C for 30 seconds) and 35 cycles. For *C5aR1* PCR, the following primers were used: RL8 (5′-GGTCTCTCCCCAGCATCATA′), RL9 (5′- GGCAACGTAGCCAAGAAAAA-3′), and RL10 (5′-GCCAGAGGCCACTTGTGTAG -3′). The PCR program included 3 steps (98°C for 15 seconds, 60.1°C for 30 seconds, 72°C for 30 seconds) and 35 cycles.

*Cmas^+/–^ F2rl3^–/–^* mice were generated by crossing *Cmas^+/–^* with the PAR4-depleted mouse strain *B6.129S4(FVB)-F2rl3^tm1.1Cgh^/Mmnc* (stock *F2rl3^tm.1.1Cgh^Cmas^tm2MhhTg15D/Bwei^*) (40). The *B6.129S4(FVB)-F2rl3^tm1.1Cgh^/Mmnc* mouse strain was obtained from the Mutant Mouse Resource and Research Center (MMRRC) at University of North Carolina at Chapel Hill (Chapel Hill, North Carolina, USA), an NIH-funded strain repository, and was donated to the MMRRC by Shaun Coughlin (UCSF, San Francisco, California, USA). The following primers were used: Par4 forward, 5′-CAGATGTTTCCTGGGCTGGGTG-3′; lacZ WT reverse, 5′-ATTGTGGGTGCCTCAGTGTCCC-3′; and Par4-KO reverse, 5′-CAGGGTTTTCCCAGTCACGACG-3′. The PCR program included 3 steps (98°C for 15 seconds, 69°C for 30 seconds, 72°C for 15 seconds) and 30 cycles.

The animals were hosted in the animal facility of the Hannover Medical School under specific pathogen–free conditions.

### Animal experiments.

Anti-C5 (BB5.1) antibody was produced as previously described (41) and applied at E4.5 and E6.5 via i.p. injection of 1,000 μg antibody in 100 μL PBS. At E8.5, female mice were sacrificed, and uteri were isolated for further processing.

Anti-GPIbα antibody (R300, Emfret Analytics) was concentrated to 2 mg/mL using Amicon Ultra-4 centrifugal filters (UFC805024) and was applied only at E4.5 or E2.5 and at E5.5 via i.p. injection of 200 μg antibody in 100 μL PBS. At E8.5, females were sacrificed, and uteri were isolated for further processing.

### Histology.

Female mice from heterozygous matings were examined daily in the early morning for the presence of a vaginal plug. The time at which the plug was discovered was considered to be day 0.5 after conception. On days 8.5 and 10.5 of gestation, the pregnant mice were sacrificed, and their uteri dissected and fixed in 4% paraformaldehyde in PBS at 4°C for 48 hours. Following fixation, the uteri were dehydrated in a graded ethanol series and embedded in paraffin wax. For histological analysis, the uteri were sectioned into 3 μm slices using a microtome, rehydrated, and stained with H&E. The slices were analyzed using a Zeiss Observer Z1 microscope equipped with a Zeiss AxioCam MRc camera. The quantification of IHC scoring was based on the means of 2 individual assessments. The statistical analyses indicated were performed using GraphPad Prism 8.4.3 (GraphPad Software). A *P* value of less than 0.05 was considered significant.

### IHC analysis.

The deparaffinized tissue was rehydrated and subjected to antigen retrieval using either a target retrieval solution (pH 6, Dako Agilent Technologies) or a proteinase K solution (50 mM Tris [pH 8.5], 1 mM EDTA, and 10 μg/mL proteinase K) for 10 minutes at 37°C. Endogenous peroxidase activity was blocked by incubating the sections in a 3% hydrogen peroxide solution in PBS at room temperature for 30 minutes. This was followed by a blocking step in 1% BSA and PBS for 30 minutes. Primary antibody incubation was performed in the blocking solution overnight at 4°C. This was followed by a 60-minute incubation with the secondary antibody at room temperature. The primary and secondary antibodies used, as well as the dilutions applied, are listed in Supplemental Table 2. All HRP-conjugated reagents were detected using a DAB (Dako) reaction and were subsequently counterstained with hematoxylin and analyzed using the aforementioned microscope setup.

### Flow cytometry.

Following euthanasia with a lethal dose of anesthetic, blood was collected from the inferior vena cava and anticoagulated with 1/10 volume of 3.8% sodium citrate. Citrated whole blood was diluted 1:25 in Tyrode’s buffer supplemented with a synthetic inhibitor of fibrin polymerization (ImmBioMed) at a final concentration of 1.5 mg/mL. Samples were stimulated with thrombin from bovine plasma (MilliporeSigma) at a final concentration of 0.01, 0.05, 0.2, or 1 IU/mL, or with ADP (Probe & go Labordiagnostica) at a final concentration of 0.5, 1, 5, or 10 μM. After agonist addition, samples were recalcified and fixed after 10 minutes with 1% BD Cytofix Fixation Buffer. Platelets were stained with anti-CD41-AF488 (BioLegend) and anti active αIIbβ3 (JON/A-PE) (Emfret Analytics) to identify platelets and assess integrin αIIbβ3 activation, respectively. Flow cytometry was performed using a BD FACSCanto II flow cytometer. Data were analyzed using FlowJo, version 10.10.0 (FlowJo). Gates were established using unstained controls, and compensation was performed using single-stained controls.

### Platelet aggregometry.

Microtiter plate–based light transmission aggregometry was performed as described by Tamang et al. (42). Briefly, citrated whole blood from 5 animals per group was pooled, and platelet-rich plasma (PRP) was prepared by gentle centrifugation at 120*g* for 15 minutes. Platelet-poor plasma (PPP) was generated by centrifugation at 3,000*g* for 10 minutes and used as a reference for maximal light transmission. PRP was adjusted to 600 × 10³ platelets/μL, and 90 μL PRP was incubated with 10 μL either Tyrode’s buffer or 10× agonist solution to achieve final concentrations of 5 IU/mL thrombin, 10 μg/mL collagen, or 10 μM ADP. Samples were incubated for 5 minutes at 37°C with shaking at 1,000 rp (Eppendorf Thermomixer), and optical density at 620 nm was measured.

### Kinetics of APC generation.

Recombinant neuraminidase from *Arthrobacter ureafaciens* (43) was conjugated to N-hydroxysuccinimide (NHS) activated beads according to the manufacturer’s instructions. As a control, NHS beads were blocked with Tris (mock). To detect neuraminidase activity, the beads were incubated in 50 mM sodium acetate buffer (pH 5.5) with Neu5Ac-pNA in a 96-well plate at 37°C, and the absorbance was measured at 405 nm.

Quantification of APC generation was performed as described previously (15). In brief, 2 nM thrombin was incubated with 1, 2, or 4 nM TM (control or ΔSia-TM, as 0.5:1, 1:1, or 2:1 TM/thrombin ratio) and various concentrations of protein C for 45 minutes at 37°C in duplicate. The reaction was then stopped by the addition of argatroban (final concentration of 4 μM). APC activity was then determined by measuring the absorbance at 405 nm following the addition of S2366 (final concentration of 0.8 mM) at 37°C. The concentration of the generated APCs was then determined using a calibration curve. To generate an APC calibration curve, various concentrations of APCs were incubated with 0.8 mM S2366 at 37°C, and the absorbance was measured at 405 nm.

### Statistics.

Statistical analyses were performed using GraphPad Prism 8.4.3 (GraphPad Software). A *P* value of less than 0.05 was considered significant. Numerical data are presented as the mean ± SD. For statistical analysis, Kruskal-Wallis test (Figures 1, 2, 5), Mann-Whitney U test (Figure 2), 2-tailed unpaired *t* test (Figure 4) and 2-way ANOVA with multiple comparisons (Figure 6) were performed.

### Study approval.

All animal experiments were approved by the Niedersaechsisches Landesamt fuer Verbraucherschutz und Lebensmittelsicherheit (LAVES) (Oldenburg, Germany; AZ33.9-42502-04-19/3150; AZ33.12-42502-04-20/3510; AZ2019/244.) and by the Institut fuer Versuchstierkunde und Zentrales Tierlaboratorium, Hannover Medical School (Hannover, Germany).

### Data availability.

Data are available in the Supporting Data Values file; or from the corresponding author upon request.

## Author contributions

AS, AT, and MA designed research studies. AS, LS, OO, MW, EA, and MA conducted experiments. AS, LS, OO, UPB, and KFS acquired data. AS, LS, OO, and MA analyzed data. AK, WMZ, BPM, KB, SW, and AT provided reagents. AS, AT, and MA wrote the manuscript. WMZ, BPM, AK, OO, and SW reviewed and edited the manuscript.

## Conflict of interest

The authors have declared that no conflict of interest exists.

## Funding support

- Deutsche Forschungsgemeinschaft (DFG, German Research Foundation) grant 409784463 (to MA) in the frame of the Research Unit “Sialic Acid as Regulator in Development and Immunity” (FOR 2953).
- UK Dementia Research Institute, through UK DRI Ltd, principally funded by the Medical Research Council grant [UKDRI-3002] (to WMZ and BPM).

## Supplementary Material

Supplemental data

Supporting data values

## Version 1 07/23/2026

In-Press Preview

## Version 2 09/15/2026

Electronic publication
