# Supplementary material for: Platelets link coagulation and complement in regulating murine placental vascular development
Source: J Clin Invest. 2026 Jul 23;136(18):e200372. doi: 10.1172/JCI200372 (PMC13574145; doi:10.1172/JCI200372)
Supplement: Supplementary file 1 [file jci-136-200372-s137.pdf]

Supplemental material

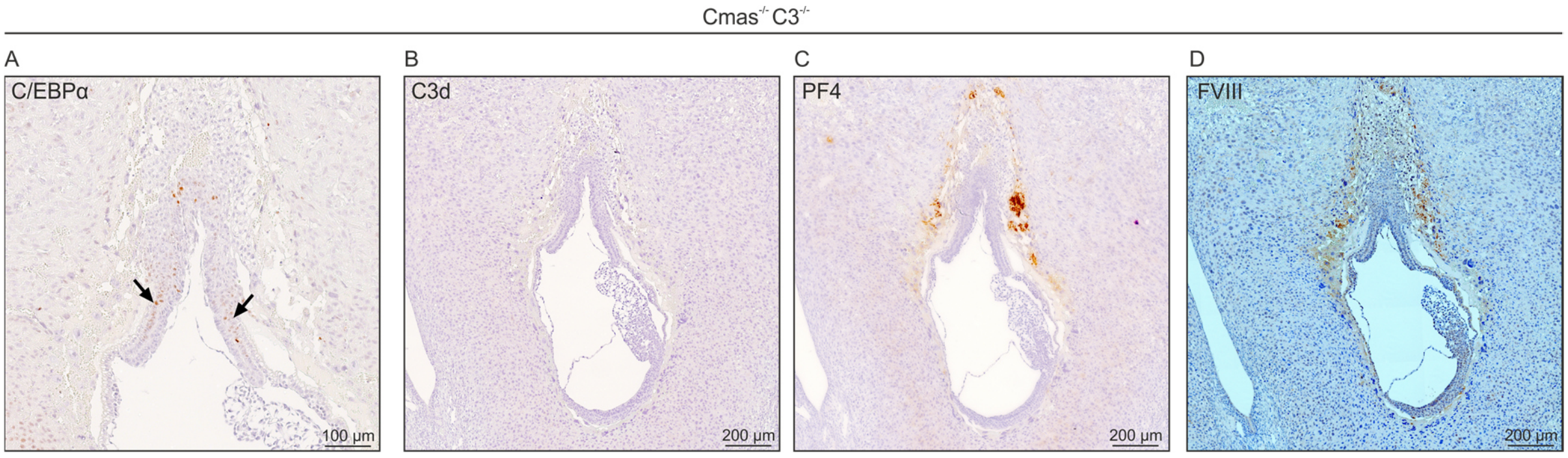

Figure S1: Loss of C3 rescues excessive thrombosis and placental development in *Cmas*$^{-/-}$ mice. (**A-C**) Representative images of C/EBPα (A), C3d (B), PF4 (C) and FVIII (D) immunostaining of *Cmas*$^{-/-}$; *C3*$^{-/-}$ placenta sections at E8.5. Arrows in A point towards the chorionic plate. For the number of individually analysed implants, see Table S1.

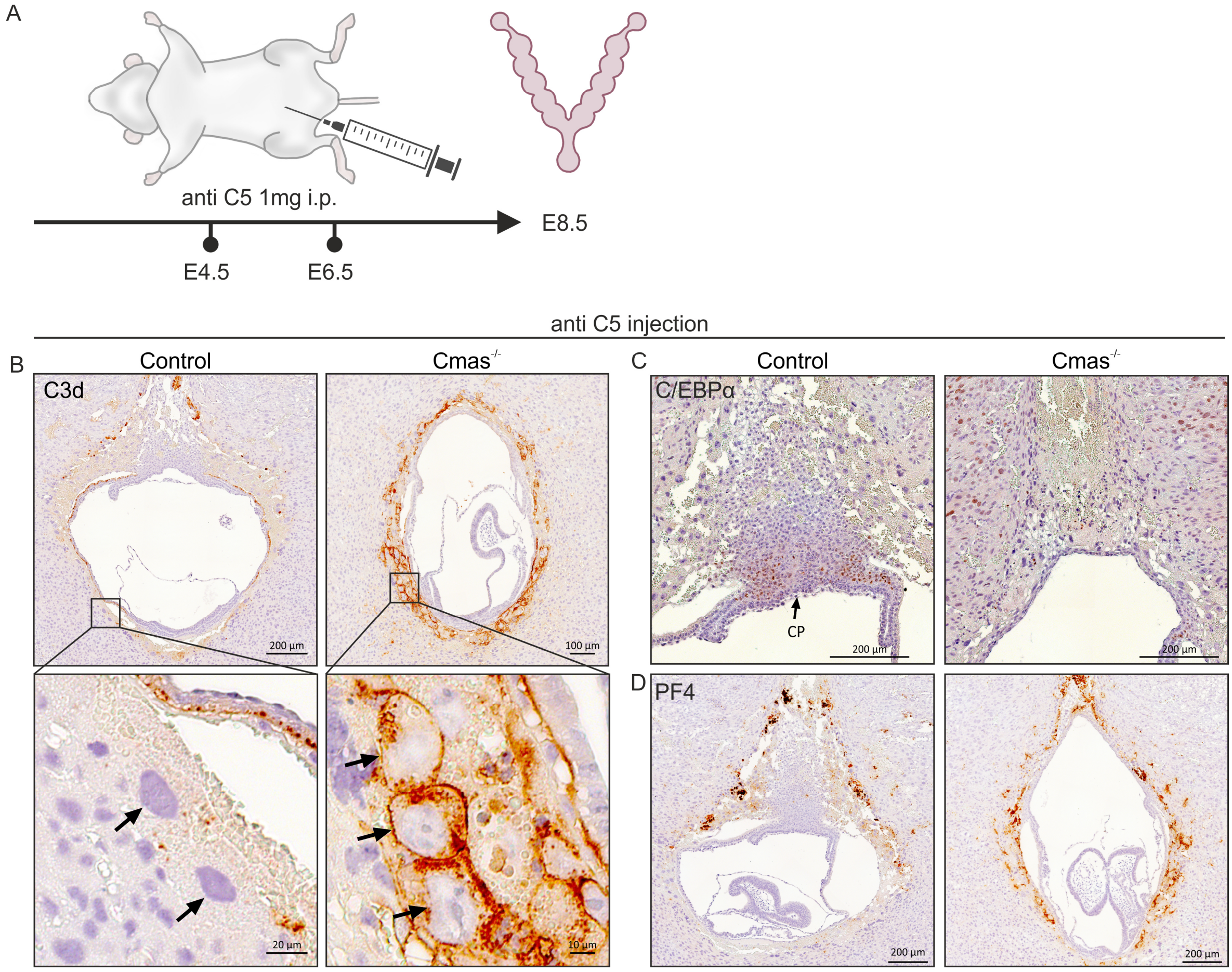

Figure S2: C5 blockade does not reduce excessive thrombosis in *Cmas*$^{-/-}$ implants. (A) Treatment regimen for C5 blockade. C5 activation was blocked by intraperitoneal injection of 1 mg anti-C5 (BB5.1) at E4.5 and E6.5 in 4 individual experiments. (**B-D**) Representative images of C3d (B), C/EBPα (C) and PF4 (D) immunostaining of *Cmas*$^{-/-}$ pregnancies under C5 blockade at E8.5. For the number of individually analysed implants, see Table S1.

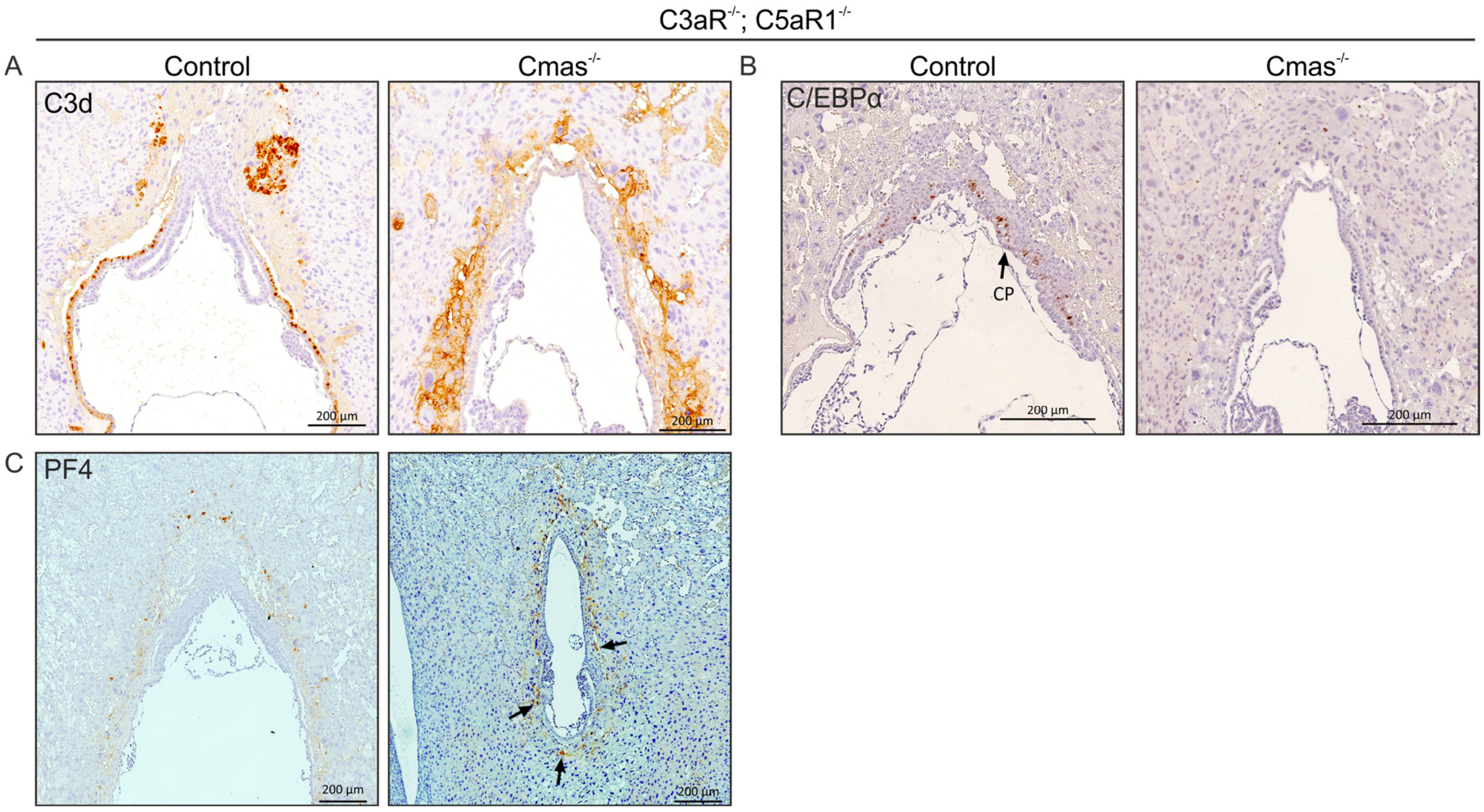

Figure S3: Impaired anaphylatoxin signaling does not rescue the phenotype of *Cmas*$^{-/-}$ mice. (**A-C**) Representative images of C3d (A), C/EBPα (B) and PF4 (C) immunostaining of *Cmas*$^{-/-}$; *C3aR*$^{-/-}$; *C5aR1*$^{-/-}$ pregnancies under C5 blockade at E8.5. For the number of individually analysed implants, see Table S1.

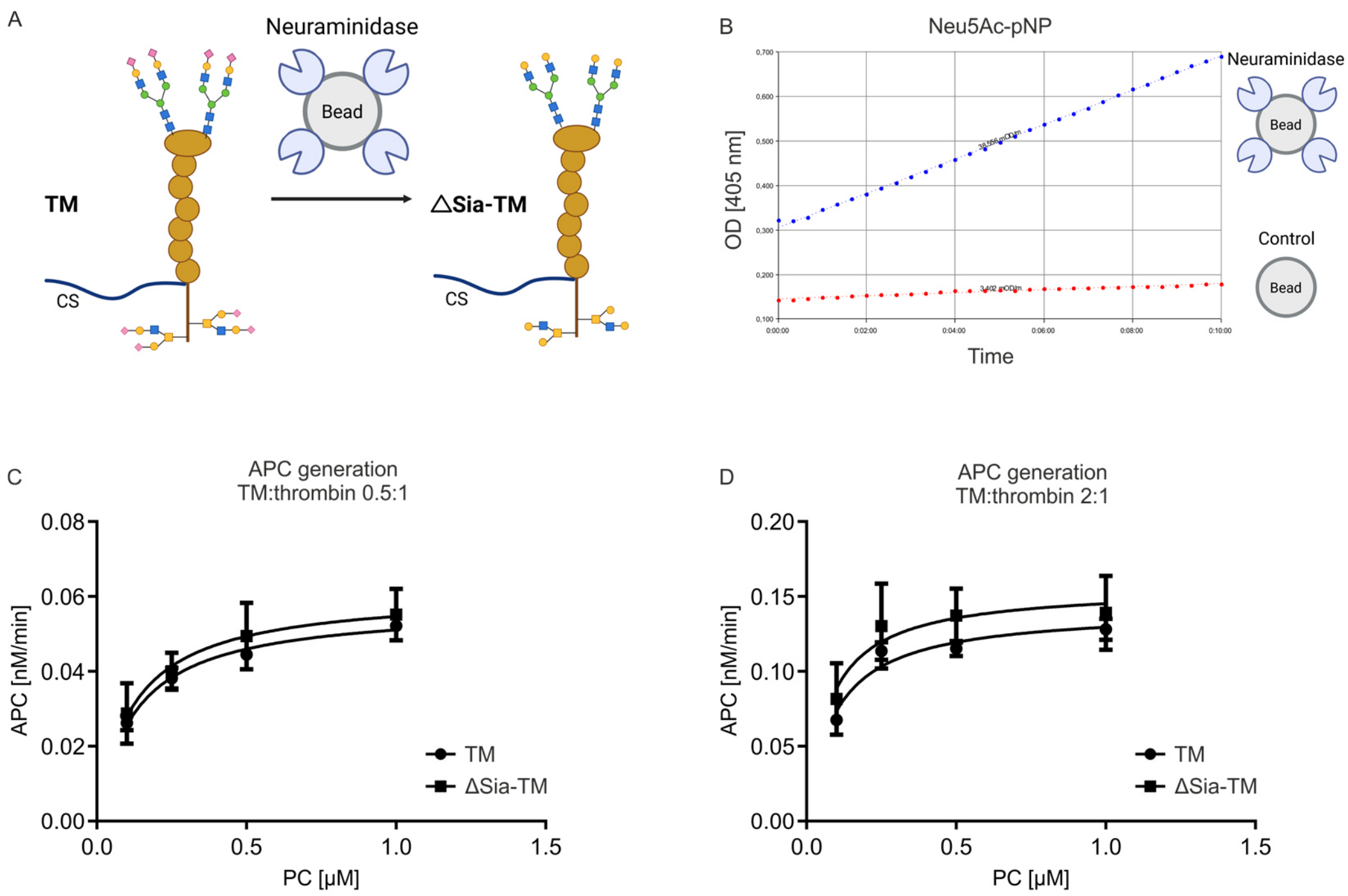

E

TM:thrombin 1:1		
	TM	ΔSia-TM
kcat	0,04739	0,05394
Std. Error kcat	0,002543	0,006772
Km	0,109	0,15
Std. Error Km	0,02349	0,06551
R²	0,9127	0,7551

TM:thrombin 0.5:1		
	TM	ΔSia-TM
kcat	0,0287	0,03062
Std. Error kcat	0,0008651	0,002898
Km	0,1245	0,1201
Std. Error Km	0,01418	0,04366
R²	0,9738	0,7806

TM:thrombin 2:1		
	TM	ΔSia-TM
kcat	0,07076	0,07817
Std. Error kcat	0,003653	0,008498
Km	0,09302	0,0767
Std. Error Km	0,02081	0,03986
R²	0,8991	0,5993

Figure S4: Generation of desialylated TM. (A) Schematic representation of bead-coupled neuraminidase induced desialylation of TM. Created in BioRender. Abeln, M. (2026) https://BioRender.com/tk2cnmv **(B)** Neuraminidase activity as measured as increase in OD [405 nm] through hydrolysis of Neu5Ac-pNP. Schematics created in BioRender. Abeln, M. (2026) https://BioRender.com/mnjr479 **(C-D)** APC generation kinetics of thrombin in 0.5:1 (C) and 2:1 (D) ratio with with TM and ΔSia-TM. Data are presented as mean ± SD of duplicates. **(E)** Enzyme kinetic parameters of APC generation by TM or ΔSia-TM in a complex with thrombin.

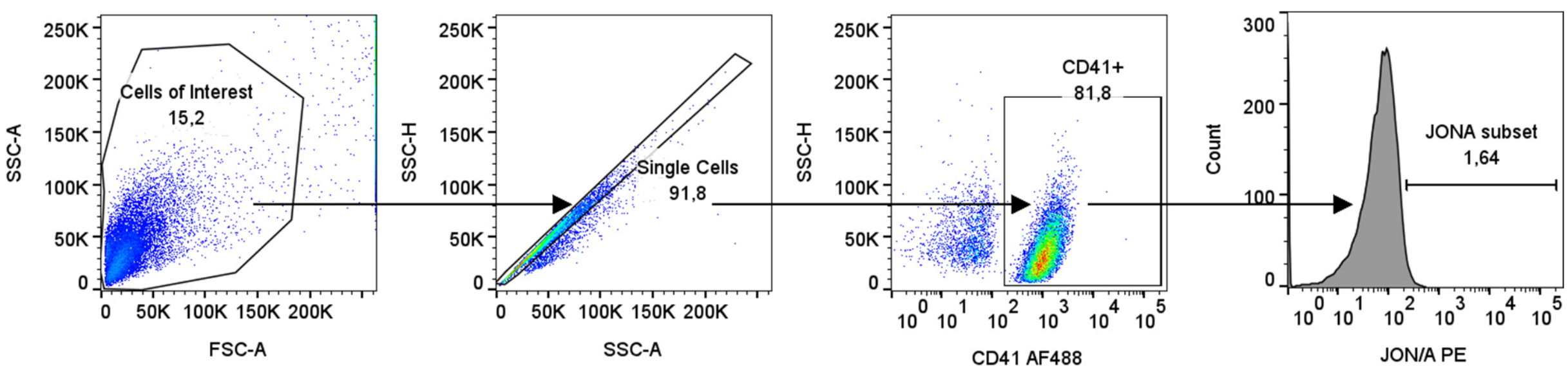

Figure S5: Gating strategy for the flow cytometry analysis of active αIIbβ3 (JON/A). After selection of cells of interest and exclusion of doublets, CD41-positive platelets were gated, and JON/A-positive events were quantified as a measure of integrin αIIbβ3 activation.

Table S1: Total numbers of analysed implants and uteri (brackets) in IHC and HE staining.

		Cmas	FVIII	PF4	C3d	Properdin	vWF	CD62P	HE	Serotonin	beta3	TM	caspase 3
Cmas	E8.5	KO	13(8)	8(5)	13(6)	9(7)		20(9)		13(6)	10 (4)	8 (3)	9 (4)
		ctrl	15(5)	5(5)	9(6)	17(7)		4(4)		9(6)	8 (4)	9 (3)	9 (4)
Cmas ; C3-/-	E8.5	KO	8(3)	5(3)	5(3)								
		ctrl	11(3)	9(3)	9(3)								
Cmas ; Par4-/-	E8.5	KO	6(4)	6(4)	6(4)	4(4)	6(5)		7(4)	6(4)	7 (4)		
		ctrl	5(4)	10(4)	10(4)	12(4)	9(5)		10(4)	5(4)	6 (4)		
Cmas anti-GPIba	2x	KO	6(2)		6(2)	4(2)			6(2)	5(2)	5 (2)		4 (2)
		ctrl	4(3)		10(3)	3(2)			10(3)	3(2)	3 (2)		7 (2)
	1x	KO											9 (4)
		ctrl											13 (5)
Cmas anti-C5	E8.5	KO		7(2)	7(2)								
		ctrl		7(2)	7(2)								
Cmas ; C3ar-/-; C5aR1-/-	E8.5	KO		8(3)	11(4)								
		ctrl		3(3)	7(4)								

Table S2: Primary and secondary antibodies used in IHC experiments.

Reagent	Host species	Label	Manufacturer	Catalog no.	Blocking reagent	Dilution
**anti-Factor VIII**	Rabbit	-	Bioss, Woburn, USA	BS-2974R	1% BSA in PBS	1:1000
**anti-Platelet factor 4**	Rat	-	R&D Systems, Minneapolis, USA	MAB595	1% BSA in PBS	1:2000
**anti-TER-119**	Rat	-	R&D Systems, Minneapolis, USA	MAB1125	1% BSA in PBS	1:5000
**anti-C3d**	Goat	-	R&D Systems, Minneapolis, USA	AF2655	1% BSA in PBS	1:2000
**anti-CD62P**	Rabbit	-	biorbyt, Cambridge, United Kingdom	BYT-ORB382723-100	5 % skimmed milk/PBS	1:1000
**anti-Ly6G**	Rat	-	BioXCell, Lebanon, USA	BP0075-1	1% BSA in PBS	1:500.000
**anti-vWF**	Rabbit	-	Dako (Agilent), Glostrup, Denmark	P 0226	5 % skimmed milk/PBS	1:100
**anti-Properdin**	Goat	-	Complement Technologies, Tyler, USA	A239	1% BSA in PBS	1:10.000
**anti-C/EBPα**	Rabbit	-	Cell Signaling Technology, Danvers, USA	8178	1% BSA in PBS	1:50
**anti-uPAR**	Rabbit	-	Bioss, Woburn, USA	bs-1927R	1% BSA in PBS	1:1500
**anti-Serotonin (5-HT)**	Rat	-	Novus Biologicals (biotechne), Centennial, USA	NB1065037SS	1% BSA in PBS	1:250
**beta3**	Rabbit	-	Novus Biologicals (biotechne), Centennial, USA	NBP2-67416	1% BSA in PBS	1:10.000
**TM**	Rabbit	-	Abcam, Cambridge, UK	ab230010	1% BSA in PBS	1:16.000
**cl caspase 3**	Rabbit	-	Cell Signaling Technology, Danvers, USA	# 9664	1% BSA in PBS	1:1000
**JON/A**	Rat	PE	Emfret Analytics, Würzburg, Germany	M023-2		
**anti-rabbit IgG**	Goat	HRP-Polymer	Vector Laboratories, Newark, USA	VEC-MP-7451-15	-	-
**anti-goat IgG**	Horse	HRP-Polymer	Vector Laboratories, Newark, USA	VEC-MP-7405-15	-	-
**anti-rat IgG**	Goat	HRP-Polymer	Vector Laboratories, Newark, USA	VEC-MP-7444-15	-	-
